# Psychometric validation of the positive youth development scale–short form in Peruvian university students

**DOI:** 10.3389/fpsyg.2026.1909136

**Published:** 2026-09-30

**Authors:** Vilma Vilca-Pareja, Rodrigo-Alejandro Ardiles-Irarrázabal, Victor Ritchar Yana-Calla, Maria Elena Rojas-Zegarra, Agueda Muñoz-Del-Carpio-Toia

**Affiliations:** 1 Escuela de Posgrado, Estudios Generales, Universidad Católica de Santa María, Arequipa, Peru; 2 Departamento de Enfermería, Universidad de Antofagasta, Antofagasta, Chile; 3 Escuela Profesional de Psicología, Universidad Católica de Santa María, Arequipa, Peru; 4 Escuela de Posgrado, Universidad Católica de Santa María, Arequipa, Peru; 5 Escuela de Posgrado, Vicerrectorado de Investigación, Estudios Generales, Universidad Católica de Santa María, Arequipa, Peru

**Keywords:** confirmatory factor analysis, measurement invariance, positive youth development, psychometric validation, university students

## Abstract

**Introduction:**

Evidence on the psychometric performance of the Positive Youth Development Scale–Short Form (PYD-SF) in Spanish-speaking university populations remains limited. This study examined evidence based on internal structure, measurement invariance across sex, internal consistency, the empirical distinctiveness of the five dimensions, and relations with purpose in life for PYD-SF scores in Peruvian university students.

**Methods:**

A cross-sectional instrumental study was conducted with 1,015 Peruvian university students aged 18 to 30 years. Confirmatory factor analysis was performed using the diagonally weighted least squares (DWLS) estimator for ordinal data. Measurement invariance across sex, internal consistency, the empirical distinctiveness of the five dimensions, and relations with purpose in life were also assessed.

**Results:**

The findings supported a correlated five-factor structure consistent with the theoretical 5Cs model. Strict measurement invariance across sex was established. Internal consistency coefficients were generally acceptable, although the categorical omega coefficient for Caring was marginal. Heterotrait–monotrait ratios remained below 0.85, and all five dimensions showed positive and statistically significant correlations with purpose in life.

**Conclusion:**

The findings provide evidence based on internal structure, internal consistency, measurement invariance across sex, the empirical distinctiveness of the five dimensions, and relations with purpose in life for PYD-SF scores in Peruvian university students. These results support their use for group-level research in comparable university populations.

## Introduction

Positive Youth Development (PYD) constitutes a contemporary theoretical paradigm that emphasizes the promotion of young people’s strengths, competencies, and social connections, rather than focusing exclusively on their deficits or problems (Lerner, 2007). From this perspective, youth are viewed as valuable resources for their communities, and not as problems to be corrected. The PYD approach maintains that if adequate conditions are created and individual and environmental assets are fostered, adolescents can develop healthy and productive life trajectories.

One of the fundamental contributions of PYD is the identification of five essential competencies, known as the 5Cs: Competence, Confidence, Character, Caring, and Connection (Kozina and Wiium, 2023). When developed in a balanced way, these dimensions enable young people to become agents of positive change for themselves and their social environment. Competence refers to positive performance in social, academic, and vocational domains; Confidence, to an internal sense of self-esteem and self-efficacy; Character, to the internalization of moral and ethical standards; Caring, to empathy and genuine concern for others; and *Connection*, to the quality of relationships with family, peers, institutions, and the community.

Although Positive Youth Development (PYD) was originally conceptualised to explain adaptive developmental processes during adolescence (Lerner et al., 2005), a growing body of evidence has demonstrated that the 5Cs model is also relevant for understanding positive development during emerging adulthood, particularly among university students (Abdul Kadir and Mohd, 2021; de Jesus et al., 2022; Shek et al., 2022; Gomez-Baya et al., 2023). The transition to higher education represents a critical developmental stage characterised by identity exploration, increasing autonomy, academic adjustment, and preparation for adult roles. Within this context, the five dimensions of the model remain theoretically meaningful, although their behavioural expression may differ from that observed during adolescence. For example, competence may be reflected in academic self-efficacy, professional development, and employability preparedness, whereas connection increasingly involves relationships with peers, faculty members, and the university community. Consequently, assessing PYD among university students has become increasingly important for understanding strengths-based developmental processes during emerging adulthood, as demonstrated by studies conducted in university populations from Brazil, Malaysia, Peru, and Spain (Abdul Kadir and Mohd, 2021; de Jesus et al., 2022; Manrique-Millones et al., 2023; Rojas Zegarra et al., 2025).

In the university context, promoting PYD attributes may contribute to students’ emotional well-being, academic resilience, and psychosocial adaptation (Shek et al., 2022). The transition to higher education involves academic and personal changes that can affect students’ adjustment and satisfaction with university life (de Jesus et al., 2022). Against this background, the PYD-SF was selected because it operationalises the five theoretically defined dimensions of PYD within a single multidimensional measure, allowing their joint assessment in university students. Its alignment with the 5Cs framework and its previous examination in both adolescent and university samples make it particularly relevant for evaluating positive development in the present population (Geldhof et al., 2014; Dvorsky et al., 2019).

The PYD-SF is a 34-item self-report measure comprising five subscales that correspond directly to the theoretical 5Cs model (Geldhof et al., 2014). Competence includes six items addressing perceived social, academic, and physical abilities; Confidence comprises six items concerning self-worth, perceived attractiveness, and positive expectations about oneself; Character includes eight items addressing behavioural conduct, social responsibility, personal values, and openness towards people from different cultural backgrounds; Caring comprises six items assessing empathy and concern for others; and Connection includes eight items concerning perceived support and relationships with family, peers, educational institutions, and the broader community. Thus, its intended multidimensional structure reflects the theoretical distinction among the five related components of positive youth development rather than treating PYD as a single undifferentiated attribute. Its coverage of personal, interpersonal, academic, and community-related domains, together with its relatively brief format, makes it a relevant candidate for large-sample research in university settings, while its performance in specific cultural and developmental contexts requires empirical examination.

However, for a measurement instrument to be effective in contexts other than the one in which it was originally created, rigorous processes of cultural adaptation and validation are required. This has motivated efforts in recent years to validate the PYD-SF in Spanish-speaking samples. For example, in Chile, the psychometric properties of the PYD constructs were explored in a sample of adolescents, and a significant impact of the Confidence and Connection dimensions on the reduction of symptoms of depression, anxiety, and stress was found (Marín-Gutiérrez et al., 2024). Although these results are relevant, it should be noted that the validation was carried out with a school-age adolescent population, not with university students, which limits its direct applicability to the university context.

Although positive youth development has been examined among Latin American university students, including in Brazil (de Jesus et al., 2022), psychometric evaluations of the PYD-SF in Spanish-speaking university populations remain scarce. In Peru, the instrument has been used previously, but comprehensive evidence regarding its psychometric performance remains limited (Manrique-Millones et al., 2023; Rojas Zegarra et al., 2025). This gap restricts the interpretation of previous findings and supports the need to examine the performance of PYD-SF scores in Peruvian university students.

Previous Peruvian studies using the PYD-SF have reported internal consistency estimates and associations with theoretically related variables (Manrique-Millones et al., 2023; Rojas Zegarra et al., 2025). However, these studies did not report a systematic evaluation of the relevance, representativeness, and comprehensibility of the item content for Peruvian university students or of the cross-cultural equivalence of the Spanish-language version. The present study addresses part of this broader psychometric gap by examining evidence based on internal structure, internal consistency, measurement invariance across sex, and relations with purpose in life. It does not directly evaluate content validity or constitute a formal cross-cultural adaptation.

Research on positive youth development in Latin America remains more limited than in Anglo-Saxon contexts, particularly regarding brief measures for university populations. Additional evidence from diverse institutions, regions, and sociocultural settings is required before broader conclusions can be drawn about the applicability of the PYD-SF across Peru or Latin America.

Although the five dimensions are theoretically related but distinct, previous research with university students has also examined whether their shared variance can be represented by a general Positive Youth Development factor (Dvorsky et al., 2019). In a bifactor model, item covariance is partitioned into a general factor shared across all items and orthogonal specific factors representing the variance associated with each dimension beyond the general factor. In the present study, this model was examined only as a secondary and exploratory analysis to assess whether interpretation of an overall score might be psychometrically defensible. It was not proposed as a replacement for the theoretically established correlated five-factor structure, which remained the primary measurement model.

Based on the above, the present study had the following objectives: (1) to evaluate the five-factor structure of the PYD-SF through confirmatory factor analysis using the diagonally weighted least squares (DWLS) estimator for ordinal data; (2) to examine measurement invariance across sex; (3) to estimate internal consistency and examine the empirical distinctiveness of the five PYD-SF dimensions; and (4) to examine relations between the PYD-SF dimensions and purpose in life; (5) as a secondary exploratory objective, to examine whether a bifactor representation supported the interpretation of a general Positive Youth Development component alongside the five specific dimensions.

Accordingly, five hypotheses were formulated. First, the PYD-SF scores were expected to be adequately represented by the correlated five-factor structure corresponding to Competence, Confidence, Character, Caring, and Connection. Second, measurement invariance across sex was expected to be supported at least at the scalar level. Third, each of the five subscale scores was expected to show acceptable internal consistency. Fourth, the five dimensions were expected to remain empirically distinguishable despite their theoretical interrelations. Finally, all five PYD-SF dimensions were expected to show positive associations with purpose in life. Because the bifactor model was examined as a secondary exploratory analysis rather than as the primary measurement model, no directional hypothesis was formulated for this analysis.

## Method

### Participants

The study was conducted using a quantitative, cross-sectional, and instrumental design, aimed at the psychometric validation of a measurement instrument (Ato et al., 2013).

To justify the adequacy of the sample size in the confirmatory factor analysis, the number of observed variables and the complexity of the proposed factorial structure were considered. The literature indicates that samples larger than 200 participants are generally considered adequate for correctly specified structural equation models (Brown, 2015; Kline, 2023). The final sample size (N = 1,015) far exceeds these criteria, which favours stability in the estimation of parameters. A non-probability convenience sample was used for their selection. Eligible participants were students with current enrolment at the participating university, aged 18 years or older, who voluntarily agreed to participate and provided informed consent. No additional exclusion criteria were applied beyond failure to meet these eligibility requirements. The final sample comprised 1,015 university students (449 men [44.2%] and 566 women [55.8%]), aged between 18 and 30 years (M = 19.6, SD = 2.0). Biological sex was self-reported by participants using a single demographic question with two response options (male/female). No information regarding gender identity or non-binary categories was collected; therefore, all multigroup analyses reported in this study refer to biological sex rather than gender.

The original dataset included 1,030 participants. Fifteen participants aged 17 years were excluded because the present analysis included only adults aged 18 years or older, resulting in a final sample of 1,015 participants. No missing data were identified in the analysed variables.

### Instruments

#### Positive youth development-SF

The Positive Youth Development–Short Form (PYD-SF; Geldhof et al., 2014) is a 34-item self-report questionnaire assessing the five dimensions of the PYD model: Competence (6 items), Confidence (6 items), Character (8 items), Caring (6 items), and Connection (8 items). All items were rated on a five-point Likert-type scale ranging from 1 (strongly disagree) to 5 (strongly agree). Subscale scores were calculated by averaging the responses to the items assigned to each dimension, resulting in scores ranging from 1 to 5. Higher scores indicate higher levels of the corresponding PYD dimension. No overall PYD score was used as the primary score because the analyses focused on the five theoretically distinct but related dimensions.

In Latin America, the PYD-SF has been the subject of various psychometric evaluations. In Chile, an adaptation showed a factorial structure consistent with the original theoretical model and satisfactory alpha coefficients in each dimension (α = 0.73–0.90) (Marín-Gutiérrez et al., 2024). In Peru, it has been used and reported acceptable values for competence (α = 0.73), confidence (α = 0.77), connection (α = 0.77), and care (α = 0.88), although the character subscale obtained a lower value (α = 0.59) (Manrique-Millones et al., 2023). A second Peruvian study reported adequate internal consistency for all dimensions, with alpha coefficients between 0.70 and 0.89 (Rojas Zegarra et al., 2025).

The Spanish-language version of the PYD-SF used in the present study was based on the version previously employed in research conducted in Chile and Peru (Manrique-Millones et al., 2023; Marín-Gutiérrez et al., 2024; Pérez-Díaz et al., 2026). Permission to use the instrument was obtained through the Cross-National Positive Youth Development Network (CN-PYD) by a member of the research team. No translation, back-translation, linguistic adaptation, or modification of item wording was conducted as part of the present study. The existing Spanish-language version was administered without adding, removing, or modifying any items. Furthermore, the present study did not include a structured expert-panel review, cognitive interviews, pilot testing specifically directed at adaptation, or quantitative content-validity assessment. Accordingly, the present research should be understood as a psychometric evaluation of an existing Spanish-language version in a large sample of Peruvian university students, rather than as a formal cross-cultural adaptation.

#### Purpose in life (PILEA)

This is a unidimensional questionnaire consisting of four Likert-type items (e.g., “My life has meaning”), with response options ranging from strongly disagree (1) to strongly agree (5) (Hill et al., 2016). The instrument was subsequently adapted into Spanish, showing adequate psychometric properties and good internal consistency (ω = 0.830) in the Spanish population (Rubio-Belmonte et al., 2022).

More recently, the abbreviated version PILEA-4 was psychometrically validated in Peruvian university students, confirming a unidimensional structure with good model fit, high reliability (ω = 0.873), and factorial invariance by sex (Vilca-Pareja et al., 2026). Additionally, research conducted in Arequipa has reported slight linguistic adaptations to adapt the items to local expressions, obtaining adequate levels of internal consistency (α = 0.820) in university samples (Rojas Zegarra et al., 2025).

### Procedure

The study was approved by the Ethics Committee of the Catholic University of Santa María (Protocol ID: 098/2023). Prior to the survey, participants were informed about the purpose of the study and their informed consent was obtained. Confidentiality and anonymity were guaranteed. Participation was entirely voluntary, and participants received no academic credit, financial compensation, gifts, or other incentives for taking part in the study. All procedures were conducted in accordance with the Declaration of Helsinki. The survey was self-administered and conducted virtually via the Microsoft Teams platform. After data collection, a database was created in Microsoft Excel 360, followed by coding and descriptive analysis in SPSS. Advanced statistical analyses were performed using RStudio. All item responses were configured as mandatory in the online survey; consequently, the final dataset contained no missing item-level data. Data quality checks were conducted to identify inconsistent response patterns, and no cases were excluded on this basis.

### Data analysis

First, descriptive statistics were calculated for each item, including the mean, standard deviation, skewness, and kurtosis, following previous methodological recommendations (Muñiz Fernández, 2018; Ferrando et al., 2022). Subsequently, the correlated five-factor model was estimated as the primary measurement model because it directly represents the theoretical structure of the five related but distinct Positive Youth Development dimensions. A bifactor model was then estimated as a secondary and exploratory analysis to examine the extent to which the common item variance could be partitioned into a general Positive Youth Development factor and five orthogonal specific factors. The bifactor model was not treated as the primary measurement model or selected solely on the basis of incremental improvements in model fit. Both models were estimated using diagonally weighted least squares (DWLS), treating all items as ordinal variables. DWLS was selected because of the ordinal nature of the items (Flora and Curran, 2004; Li, 2016; Ferrando et al., 2022). The same non-robust DWLS estimator was used throughout the measurement-invariance analyses. Model fit was evaluated using the comparative fit index (CFI), Tucker–Lewis index (TLI), root mean square error of approximation (RMSEA), and standardised root mean square residual (SRMR). As general interpretative guidelines, CFI and TLI values ≥ 0.90 were considered acceptable and values ≥ 0.95 indicative of good fit, whereas RMSEA and SRMR values ≤ 0.08 were considered acceptable (Hu and Bentler, 1999; Brown, 2015). These indices were interpreted jointly rather than as rigid independent decision rules.

The correlated five-factor model was initially estimated without correlated residuals. Residual covariances were considered only when two conditions converged: a modification index indicating local dependence and a substantively plausible source of item-specific shared variance not represented by the broader latent factor (Brown, 2015). Six within-factor item pairs met both conditions. Items 3 and 8 shared a sports-specific aspect of Competence; items 12 and 13 were closely redundant statements of global self-acceptance; items 19 and 20 shared the narrow referent of openness towards people from other racial or cultural groups; items 23 and 26 used closely related sadness-based reactions to others’ adversity; items 27 and 30 shared the institutional referent of school or university support; and items 33 and 34 referred to the same supportive-friendship context. These covariances were therefore interpreted as localised item-specific dependence beyond the variance explained by the corresponding latent factor, rather than as additional relations between constructs. They were introduced *post hoc* and were not selected solely because they improved model fit. The same residual-covariance specification was retained in the bifactor and measurement-invariance models to preserve consistency across model specifications. Next, measurement invariance across sex was examined using the measEq.syntax function from the semTools package in R. All 34 items were specified as ordered variables (ordered = TRUE), and the models were estimated using DWLS with theta parameterization. Ordinal multigroup identification followed Wu and Estabrook’s (2016) procedure (Wu and Estabrook, 2016). The configural model imposed no equality constraints across groups beyond those required for model identification. Equality constraints were then introduced sequentially: item thresholds in the threshold-invariance model; thresholds and factor loadings in the metric-invariance model; thresholds, factor loadings, and item intercepts in the scalar-invariance model; and thresholds, factor loadings, item intercepts, and residual variances in the strict-invariance model. Under theta parameterization, scalar invariance concerns equality of thresholds, factor loadings, and intercepts of the continuous latent response variables underlying the observed ordinal items, whereas strict invariance additionally constrains their residual variances across groups. Measurement invariance was evaluated from changes in model fit. Decreases in CFI ≤ 0.010 and increases in RMSEA ≤ 0.015 were considered acceptable. For SRMR, increases ≤ 0.030 were applied when testing equality of factor loadings, whereas increases ≤ 0.010 were applied to invariance tests involving thresholds, intercepts, or residual variances (Cheung and Rensvold, 2002; Chen, 2007).

Subsequently, validity evidence based on relations with another variable was evaluated. Internal consistency was estimated using ordinal alpha (αord), categorical omega3 (ω3), and composite reliability (CR). Ordinal alpha and omega3 were obtained from the alpha.ord and omega3 outputs, respectively, using the reliability() function of the semTools package applied to the confirmatory factor analysis model with ordered indicators. Composite reliability was calculated using the compRelSEM() function from semTools. The empirical distinctiveness of the five PYD-SF dimensions was examined using the heterotrait–monotrait ratio (HTMT), calculated with the htmt() function from semTools, with all indicators specified as ordered variables and polychoric correlations used in the estimation. HTMT values below 0.85 were interpreted as indicating that the dimensions remained empirically distinguishable despite their expected theoretical associations (Henseler et al., 2015). In addition, latent mean differences were estimated to explore variations between groups while controlling for measurement error.

All analyses were performed in RStudio, using the lavaan (version 0.6–19) and semTools (version 0.5–7) packages.

## Results

### Descriptive analysis

Table 1 presents the descriptive statistics for the items. Overall, the means fell within the mid-to-high range, and no extreme deviations were observed in the measures of skewness and kurtosis. Given the ordinal nature of the Likert-type responses, the items were treated as ordinal variables in the subsequent analyses (Flora and Curran, 2004).

**Table 1:** Descriptive statistics and factor loadings for the PYD-SF items.

Items	Min	Max	M	SD	g_1_	g_2_	λ	SE	95% CI	R^2^
Factor 1: competence										
1. Tengo muchos amigos	1	5	3.37	0.94	−0.32	−0.31	0.614	0.022	[0.572, 0.657]	0.377
2. Me va muy bien en mi clase en el colegio/la universidad	1	5	3.58	0.77	−0.45	0.35	0.651	0.023	[0.607, 0.696]	0.424
3. Soy mejor que otros de mi edad en los deportes	1	5	2.86	1.01	0.06	−0.36	0.449	0.027	[0.396, 0.503]	0.202
7. Soy tan inteligente como los demás chicos de mi edad	1	5	3.61	0.88	−0.36	−0.06	0.663	0.022	[0.619, 0.707]	0.440
8. Podría hacerlo bien en casi cualquier actividad deportiva nueva	1	5	3.31	1.05	−0.38	−0.45	0.554	0.024	[0.506, 0.601]	0.307
9. Soy popular entre las personas de mi edad	1	5	2.89	0.92	−0.06	−0.18	0.684	0.020	[0.645, 0.722]	0.468
Factor 2: confidence										
4. Estoy contento conmigo mismo la mayor parte del tiempo	1	5	3.46	0.90	−0.43	−0.11	0.781	0.014	[0.753, 0.808]	0.610
6. Me gusta mucho como me veo	1	5	3.46	0.92	−0.47	0.04	0.804	0.013	[0.779, 0.829]	0.646
10. Me considero una persona atractivo/a	1	5	3.40	0.91	−0.51	0.34	0.734	0.016	[0.703, 0.766]	0.539
12. Estoy muy feliz de ser como soy	1	5	3.68	0.87	−0.58	0.41	0.862	0.012	[0.838, 0.886]	0.743
13. En general, me gusta como soy	1	5	3.69	0.86	−0.55	0.26	0.875	0.011	[0.854, 0.896]	0.766
14. Cuando sea adulto, estoy seguro de que tendré una buena vida	1	5	3.91	0.78	−0.42	0.20	0.668	0.022	[0.626, 0.711]	0.446
Factor 3: character										
5. Casi nunca hago cosas que sé que no debo hacer	1	5	3.59	0.81	−0.28	−0.33	0.315	0.032	[0.253, 0.378]	0.099
11. Normalmente actúo como se supone que debo hacerlo	1	5	3.70	0.70	−0.66	1.16	0.612	0.025	[0.563, 0.660]	0.375
15. Contribuyo a que el mundo sea un mejor lugar para vivir	1	5	3.67	0.78	−0.53	0.49	0.669	0.022	[0.627, 0.712]	0.448
16. Me responsabilizo de mis acciones cuando cometo un error o me meto en problemas	1	5	4.06	0.68	−0.52	0.86	0.663	0.022	[0.619, 0.707]	0.440
17. Doy tiempo y dinero para mejorar la vida de las personas	1	5	3.36	0.84	−0.31	−0.02	0.494	0.027	[0.442, 0.546]	0.244
18. Hago lo correcto incluso si los demás no están de acuerdo	1	5	3.81	0.68	−0.54	0.81	0.705	0.020	[0.665, 0.744]	0.497
19. Disfruto estar con personas de una raza o cultura diferente	1	5	3.96	0.78	−0.75	1.31	0.538	0.026	[0.488, 0.588]	0.289
20. Me agrada saber mucho sobre personas de otras razas o culturas	1	5	4.02	0.77	−0.87	1.59	0.576	0.025	[0.526, 0.625]	0.332
Factor 4: caring										
21. Cuando veo que se están aprovechando de alguien, quiero ayudarlo/a	1	5	4.05	0.75	−1.12	2.72	0.825	0.020	[0.786, 0.865]	0.681
22. Cuando veo que están molestando a alguien, me siento triste por esa persona	1	5	3.87	0.82	−0.89	1.44	0.845	0.016	[0.813, 0.876]	0.714
23. Cuando veo que una persona está herida o molesta, me siento triste por esa persona	1	5	3.58	0.95	−1.11	1.17	0.445	0.023	[0.400, 0.491]	0.198
24. Me afecta cuando le pasan cosas malas a otros	1	5	3.60	0.84	−0.46	0.32	0.666	0.022	[0.623, 0.709]	0.444
25. Siento pena por otras personas que no tienen lo que yo tengo	1	5	3.52	0.86	−0.35	−0.04	0.595	0.024	[0.548, 0.643]	0.354
26. Me entristece ver a una persona que no tiene amigos	1	5	3.45	0.98	−0.73	0.33	0.396	0.028	[0.341, 0.451]	0.157
Factor 5: connection										
27. Recibo mucho apoyo en mi colegio/universidad	1	5	3.41	0.81	−0.52	0.55	0.599	0.024	[0.553, 0.645]	0.359
28. Soy un miembro útil e importante de mi familia	1	5	3.04	1.17	−0.84	−0.85	0.331	0.027	[0.277, 0.384]	0.110
29. Me siento un miembro útil e importante de mi comunidad	1	5	3.49	0.83	−0.30	0.16	0.753	0.019	[0.715, 0.791]	0.567
30. Los profesores en el colegio/la universidad me incentivan a ser lo mejor que puedo ser	1	5	3.68	0.82	−0.64	0.76	0.604	0.024	[0.558, 0.651]	0.365
31. Tengo conversaciones profundas con mis padres	1	5	3.37	1.08	−0.50	−0.32	0.591	0.022	[0.548, 0.634]	0.349
32. Los adultos en mi pueblo/ciudad/comunidad escuchan mi opinión	1	5	3.24	0.94	−0.36	−0.11	0.647	0.020	[0.608, 0.686]	0.419
33. Mis amigos se preocupan por mí	1	5	3.77	0.82	−0.73	0.95	0.634	0.023	[0.588, 0.680]	0.402
34. Considero que tengo buenos amigos.	1	5	3.92	0.84	−0.71	0.66	0.598	0.025	[0.550, 0.647]	0.358

Min: minimum value; Max: maximum value; M: mean; SD: standard deviation; g_1_: skewness; g_2_: kurtosis; λ: standardized factor loading; SE: standard error; CI: 95% confidence interval; R^2^: explained variance. Item wording is presented in the Spanish version administered to participants.

### Confirmatory factor analysis

The initial correlated five-factor model without residual covariances yielded χ^2^(517) = 5366.95, CFI = 0.950, TLI = 0.946, SRMR = 0.078, and RMSEA = 0.096 (95% CI [0.094, 0.099]). Although the comparative fit index, Tucker–Lewis index, and standardized root mean square residual were within the prespecified criteria, the root mean square error of approximation exceeded 0.080, indicating mixed evidence of model fit. After introducing the six within-factor residual covariances described in the Data Analysis section, the final correlated five-factor model yielded χ^2^(511) = 2903.67, CFI = 0.975, TLI = 0.973, SRMR = 0.061, and RMSEA = 0.068 (95% CI [0.066, 0.070]). Considered jointly, these indices supported the final correlated five-factor structure. Because the residual covariances were introduced *post hoc*, they were interpreted cautiously as localised item-specific dependence rather than as relations between the latent constructs. Complete results for both model specifications are presented in Table 2.

**Table 2:** Fit indices for the confirmatory factor models.

Models	Fit indices					RMSEA 95% CI		
	χ^2^	df	CFI	TLI	SRMR	RMSEA	LL	UL
Initial correlated five-factor model without residual covariances	5366.95	517	0.950	0.946	0.078	0.096	[0.094]	[0.099]
Final correlated five-factor model with six residual covariances	2903.67	511	0.975	0.973	0.061	0.068	[0.066]	[0.070]
Bifactor model	3553.06	487	0.968	0.964	0.069	0.079	[0.076]	[0.081]

χ^2^: chi-square; df: degrees of freedom; CI: confidence interval; LL: lower limit, UL: upper limit.

Furthermore, a bifactor model was estimated to examine whether the covariance among the five dimensions could be explained by a general Positive Youth Development factor while retaining the five specific domains. The same residual-covariance specification used in the correlated five-factor model was retained. Although the model showed acceptable overall fit, its fit was poorer than that of the correlated five-factor model (Table 2). Additional indices from the bifactor model are reported in Table 3. The general factor accounted for 58.7% of the common variance, while the item-level proportions attributable to the general factor ranged from 0.057 to 0.999 and are reported in Supplementary Table S1. Reliability attributable uniquely to the specific dimensions ranged from 0.175 to 0.616. Taken together, the moderate proportion of common variance explained by the general factor, the wide variation across items, and the heterogeneous specific-dimension reliability estimates provide exploratory evidence of a general Positive Youth Development component, but do not support essential unidimensionality or an unequivocal interpretation of a total score. Accordingly, the correlated five-factor model was retained as the primary representation of the internal structure.

**Table 3:** Reliability and empirical distinctiveness of the five PYD-SF dimensions.

Factors	αord	CR	ω3	HTMT				
				1	2	3	4	5
1. Competence	0.786	0.775	0.700	_				
2. Confidence	0.901	0.908	0.894	0.830	_			
3. Character	0.805	0.798	0.718	0.365	0.550	_		
4. Caring	0.812	0.805	0.690	0.195	0.117	0.551	_	
5. Connection	0.823	0.816	0.749	0.672	0.671	0.573	0.369	_

αord = ordinal alpha coefficient; ω3 = categorical omega3 coefficient; CR = composite reliability; HTMT = heterotrait–monotrait ratio; ωH = omega hierarchical; ECV = explained common variance; PUC = percentage of uncontaminated correlations; H = construct replicability; ωHS = omega hierarchical subscale. For the general factor, ωH = 0.815, ECV = 0.587, PUC = 0.820, and H = 0.929. The ωHS coefficients were 0.175 for Competence, 0.276 for Confidence, 0.296 for Character, 0.616 for Caring, and 0.189 for Connection. Item-level IECV values are presented in Supplementary Table S1.

Standardized factor loadings ranged from 0.315 to 0.875 across the five dimensions. Although most items showed moderate to high loadings, items 5, 23, 26, and 28 showed comparatively weak factorial representation (R^2^ = 0.099, 0.198, 0.157, and 0.110, respectively). These items were retained to preserve the original 34-item configuration of the PYD-SF and comparability with previous applications, rather than solely because their standardized loadings exceeded a predetermined threshold. Their factorial performance should be examined further in future studies. Detailed information on each item’s wording, assigned factor, standardized factor loading, standard error, 95% confidence interval, and explained variance (R^2^) is provided in Table 1 and Figure 1.

**Figure 1:**
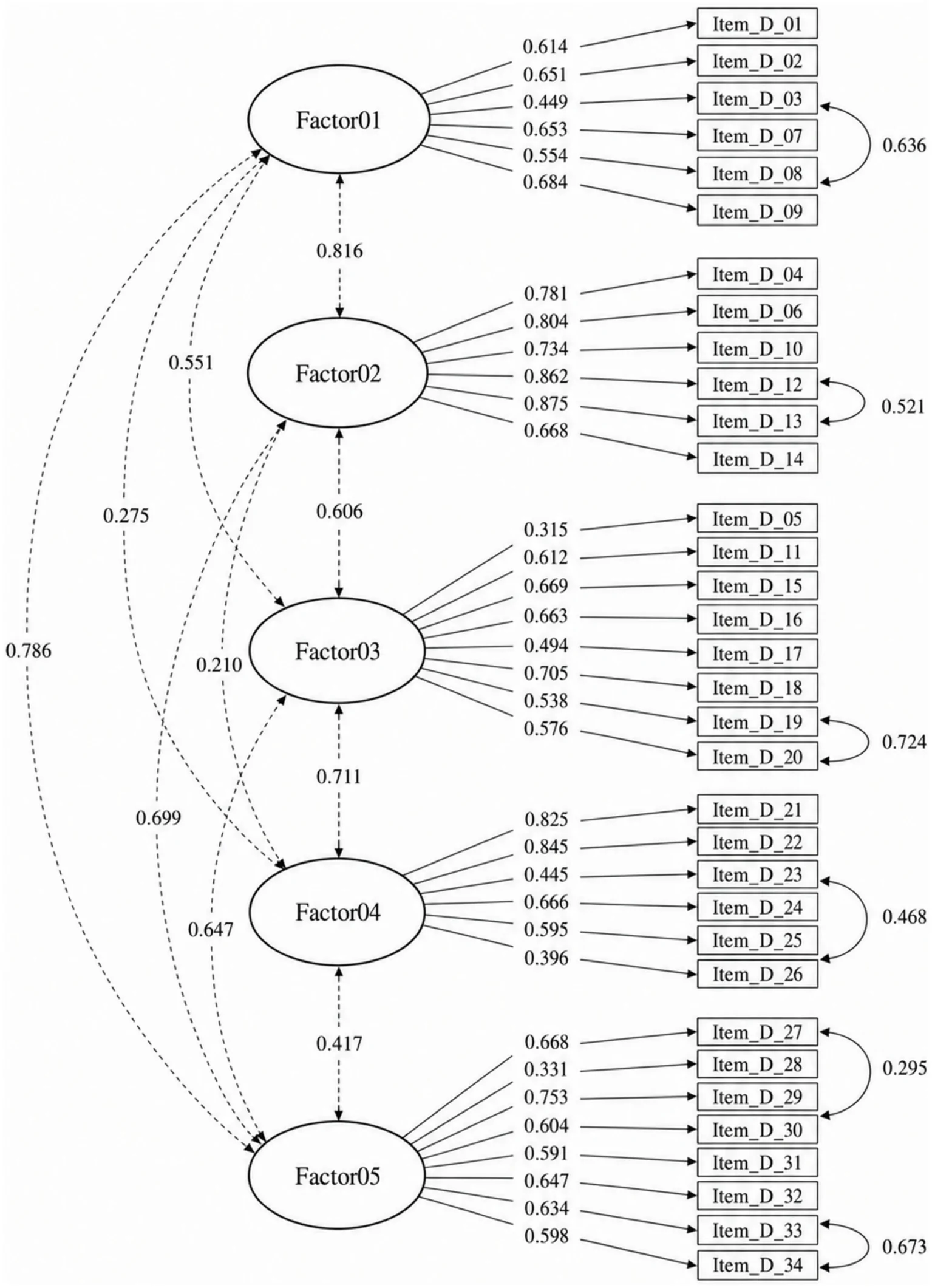
Factor loadings from the confirmatory factor analysis of the 5Cs.

### Factorial invariance

A multigroup factorial invariance analysis by sex was performed using the diagonally weighted least squares estimator. The six residual covariances specified in the final correlated five-factor model -between items 3–8, 12–13, 19–20, 23–26, 27–30, and 33–34- were retained across all measurement-invariance models. As shown in Table 4, invariance was sustained at all levels. The configural model showed adequate fit, indicating equivalent factor structure in men and women. When equality constraints were progressively imposed on thresholds, factor loadings, item intercepts, and residual variances, changes in model fit were small and remained within the prespecified criteria (Table 4). After scalar and strict invariance were supported, latent means were compared using women as the reference group. Men showed lower latent means in Competence (estimated difference = −0.504, standard error = 0.081, *p* < 0.01; absolute Glass’s delta = 0.476) and Confidence (estimated difference = −0.338, standard error = 0.072, *p* < 0.01; absolute Glass’s delta = 0.312). Men showed a higher latent mean in Character (estimated difference = 0.306, standard error = 0.072, *p* < 0.01; absolute Glass’s delta = 0.316). No statistically significant differences were observed in Caring or Connection. Effect sizes were calculated using the standard deviation of the female reference group and are reported as absolute magnitudes.

**Table 4:** Invariance of the 5Cs according to sex.

Models	χ^2^	df	CFI	SRMR	RMSEA	ΔCFI	ΔSRMR	ΔRMSEA
Sex (men vs women)								
Configural	3529.03	1,024	0.975	0.067	0.069	-	-	-
Threshold	3576.95	1,092	0.976	0.067	0.067	0.001	0.000	−0.002
Metric	3712.33	1,121	0.975	0.067	0.068	−0.001	0.000	0.001
Scalar	3927.94	1,150	0.973	0.067	0.069	−0.002	0.000	0.001
Strict	4329.32	1,184	0.969	0.070	0.072	−0.004	0.003	0.003

ΔCFI: Change in comparative fit index; ΔSRMR: Change in standardized root mean square residual; ΔRMSEA: Change in root mean square error of approximation.

### Reliability and empirical distinctiveness of the five dimensions

Internal consistency estimates are presented in Table 3. Ordinal alpha coefficients ranged from 0.786 to 0.901, whereas categorical omega coefficients ranged from 0.690 to 0.894. The categorical omega coefficient for Caring was marginal and should therefore be interpreted cautiously. Composite reliability coefficients ranged from 0.775 to 0.908. These coefficients provide evidence of internal consistency but should not be interpreted as evidence of temporal stability or score accuracy. The bifactor-specific reliability and dimensionality indices are also reported in Table 3 and were interpreted as exploratory rather than as unequivocal support for an overall Positive Youth Development score. Regarding the empirical distinctiveness of the five dimensions, heterotrait–monotrait ratios ranged from 0.117 to 0.830 and remained below the criterion of 0.85 (Henseler et al., 2015). The highest ratio was observed between Competence and Confidence (0.830), indicating considerable overlap while supporting their treatment as empirically distinguishable dimensions according to the selected criterion (Table 3).

### Validity evidence based on relations with another variable

Regarding validity evidence based on relations with another variable, all five PYD-SF dimensions showed positive and statistically significant correlations with purpose in life, ranging from 0.192 for Caring to 0.562 for Confidence (*p* < 0.01). The correlations were 0.488 for Competence, 0.562 for Confidence, 0.387 for Character, 0.192 for Caring, and 0.464 for Connection (Table 5). According to Cohen’s (1992) conventional criteria, the associations were small for Caring, moderate for Competence, Character, and Connection, and large for Confidence. These theoretically consistent associations provide validity evidence based on relations with another variable. Because purpose in life is theoretically related to, but does not measure the same constructs as, the five PYD-SF dimensions, these associations should not be interpreted as a direct test of convergent validity.

**Table 5:** Correlations between the five PYD-SF dimensions and purpose in life.

Variables	M	SD	1	2	3	4	5	6
1. Competence	3.3	0.6	–					
2. Confidence	3.6	0.7	0.648^**^	–				
3. Character	3.8	0.5	0.355^**^	0.453^**^	–			
4. Caring	3.7	0.6	0.170^**^	0.123^**^	0.450^**^	–		
5. Connection	3.5	0.6	0.538^**^	0.554^**^	0.452^**^	0.299^**^	–	
6. Purpose in life	3.9	0.7	0.488^**^	0.562^**^	0.387^**^	0.192^**^	0.464^**^	–

M: Mean; SD: standard deviation. **p < 0.01.

## Discussion

The present study evaluated the five-factor structure of the PYD-SF through confirmatory factor analysis in university students from a private university in Arequipa, Peru. The findings supported the correlated five-factor model as an acceptable representation of the internal structure. Within the scope of the evidence examined, these results support the interpretation of PYD-SF subscale scores for group-level research in this sample and comparable university populations.

The improved fit obtained after specifying six within-factor residual covariances should not be interpreted as evidence of additional relations between the latent constructs. These parameters reflect localised item-specific dependence associated with narrow shared referents or closely overlapping item content, as detailed in the Data Analysis section. Because they were introduced *post hoc*, their replicability should be examined in independent samples, and they should not be considered modifications of the theoretical five-factor structure. Accordingly, the substantive interpretation remains centred on the five correlated Positive Youth Development dimensions.

These findings can be considered alongside psychometric evidence obtained from university samples in other regions. In a multi-university sample of college students in the United States, Dvorsky et al. (2019) compared one-factor, correlated five-factor, second-order, and bifactor representations and found support for the bifactor model, including measurement invariance across sex. In an Asian university context, Abdul Kadir and Mohd (2021) applied the PYD-SF among emerging adults from Malaysian universities and reported satisfactory internal consistency across the five dimensions and theoretically coherent associations with well-being. Collectively, these studies support continued examination of the 5Cs during emerging adulthood, although differences in samples, cultural settings, and model specifications preclude direct comparisons.

The present findings add evidence from a Peruvian university sample to previous research examining the five-factor structure of the PYD model in Latin America (Marín-Gutiérrez et al., 2024). However, because participants were recruited from a single private university, the results do not establish the applicability or cultural relevance of the PYD-SF across Peru, Latin America, or other non-Anglo-Saxon contexts. Further studies involving diverse institutions, regions, and sociocultural groups are required before drawing broader conclusions.

Consistent with contemporary validity theory, the present study provides evidence supporting specific interpretations of PYD-SF scores, particularly evidence based on internal structure and relations with another variable, together with internal consistency, the empirical distinctiveness of the five dimensions, and measurement invariance across sex.

The present findings add to research examining the PYD framework during emerging adulthood. Although the PYD-SF was originally developed for adolescent populations (Geldhof et al., 2014), the correlated five-factor model showed acceptable fit in this university sample. However, this finding does not by itself establish the developmental appropriateness or cultural equivalence of the items. Interpretations should therefore remain limited to the PYD-SF subscale scores and the population evaluated in this study. Future research should directly examine item relevance and comprehension among university students from diverse Peruvian institutions and sociocultural settings.

The observed heterogeneity in standardized factor loadings suggests that some items represented their assigned dimensions less strongly in this university sample. The lowest loading was observed for item 5, “I hardly ever do things I know I should not do” (Character), whose negative wording and emphasis on behavioural restraint may capture only a narrow aspect of Character. Item 28, “I am a useful and important member of my family” (Connection), may be influenced by differences in family roles, living arrangements, geographical mobility, and economic independence during emerging adulthood. Items 26, “It makes me sad to see a person who has no friends” (Caring), and 23, “When I see a person who is hurt or upset, I feel sorry for that person” (Caring), reflect specific emotional reactions that may not encompass the broader empathic concern represented by the Caring dimension. Finally, item 3, “I am better than others my age at sports” (Competence), may have reduced relevance for university students whose competence is more strongly expressed through academic, professional, or autonomous functioning. These interpretations are exploratory and do not establish developmental or cultural causes for the lower loadings. Rather, they identify item content that should be examined through cognitive interviews and formal content-validity studies before considering any modification of the instrument. In the present study, the bifactor model was examined only as a secondary and exploratory analysis, following previous research that evaluated this structure in university students (Dvorsky et al., 2019). This specification assumes that the general factor is orthogonal to the five specific factors; therefore, it should not be interpreted as indicating that a general Positive Youth Development factor is correlated with the five dimensions. Rather, its purpose was to separate the common variance shared across all items from the variance uniquely associated with each dimension. Because the bifactor model showed poorer fit than the correlated five-factor model and produced heterogeneous specific-dimension indices, it was not retained as the primary representation of the instrument and does not provide sufficient support for interpreting an overall score. One-factor, higher-order, and exploratory structural equation models were not estimated. Future studies could examine alternative specifications, including exploratory structural equation modelling and bifactor exploratory structural equation modelling, to investigate possible sources of residual multidimensionality and potential inflation of factor correlations (Ferrando et al., 2022).

At the content level, the five dimensions may not be equally represented in emerging adult university students. Competence includes academic, sports, cognitive, and peer-popularity indicators, some of which may have reduced developmental salience in this population. Confidence is primarily represented by self-regard items, although the item referring to future adulthood may be developmentally incongruent for respondents who are already adults. Character and Caring include conceptually relevant indicators of moral conduct, social responsibility, empathy, and concern for others; however, their content coverage was not independently assessed. Connection encompasses family, peer, educational, and community relationships, whose relevance may vary according to living arrangements, mobility, economic independence, and autonomy. These considerations are interpretive and should not be regarded as evidence of content validity.

Factorial invariance across sex reached the strict level, enabling latent mean comparisons between men and women. The choice of the DWLS estimator reflected the ordinal nature of the items. This approach is consistent with current recommendations for confirmatory factor analysis with Likert-type data (Lloret-Segura et al., 2014; Ferrando et al., 2022).

Evidence based on relations with another variable was provided by the positive and statistically significant associations between all five PYD-SF dimensions and purpose in life, consistent with previous evidence linking positive youth development with purpose in life (Pérez-Díaz et al., 2024). Confidence showed the strongest association, followed by Competence, Connection, Character, and Caring. The comparatively strong association observed for Confidence is theoretically coherent because this dimension involves self-worth, self-efficacy, and positive beliefs about one’s capacities (Lerner et al., 2005), whereas purpose in life involves direction, agency, and personal meaning (McKnight and Kashdan, 2009; Hill et al., 2016). These personal resources may facilitate the formulation of coherent goals and the intentional organisation of one’s life trajectory during emerging adulthood.

Caring showed the weakest association with purpose in life. Within the 5Cs model, Caring primarily reflects empathy, sympathy, and concern for others (Lerner et al., 2005), whereas purpose in life is more directly related to personal direction, meaning, and goal orientation (McKnight and Kashdan, 2009; Hill et al., 2016). Therefore, the smaller association is theoretically plausible and suggests that these constructs are related but conceptually distinct. Overall, the observed pattern provides theoretically consistent validity evidence based on relations with another variable, rather than constituting a direct test of convergent validity.

The psychometric evaluation of the PYD-SF in Peruvian university students has potential implications for higher education. Nevertheless, any proposed use should be limited to the sources of evidence examined in the present study.

Based on the evidence obtained in the present study, the PYD-SF appears appropriate for group-level research examining positive youth development among university students and its associations with psychosocial outcomes such as purpose in life, well-being, academic adjustment, resilience, and mental health indicators. The instrument may also be useful for descriptive institutional monitoring of strengths-based developmental attributes within university programmes focused on socioemotional development, mentoring, student engagement, or community participation. However, the present findings do not support the use of PYD-SF scores for individual diagnosis, clinical assessment, admission or selection decisions, high-stakes classification, interpretation based on cut-off scores, individual feedback reports, comparisons between institutions, or evaluation of intervention effectiveness without longitudinal or experimental evidence. These applications require additional sources of validity evidence and more comprehensive interpretative guidelines (Messick, 1995; American Educational Research Association, American Psychological Association, and National Council on Measurement in Education, 2014).

A key methodological strength is the large sample size, which supports the stability of the estimated parameters and provides adequate statistical power for demanding multigroup analyses, such as measurement-invariance testing. Demonstrating strict measurement invariance across sex constitutes another important strength.

Among the main limitations is the use of convenience sampling at a single private university in Arequipa, which restricts the generalisation of the results to other institutional contexts in the country. Likewise, the cross-sectional design prevents the establishment of causal relationships between the variables studied, so the results must be interpreted within the specific framework of the sample and the moment evaluated. Similarly, the online administration of the instrument may have generated biases in the responses and highlights the need for more rigorous quality control procedures. Furthermore, the present study used an existing Spanish-language version of the PYD-SF without conducting a new formal cross-cultural adaptation process. Specifically, the study did not include translation or back-translation, cognitive interviews, structured expert-panel assessment, pilot testing specifically directed at adaptation, or quantitative content-validity assessment. Consequently, future studies should conduct a formal cross-cultural adaptation following established methodological guidelines (Beaton et al., 2000; Muñiz et al., 2013), including cognitive interviews, structured expert judgement, adaptation-specific pilot testing, and quantitative assessment of content validity, to strengthen evidence regarding the linguistic, cultural, and developmental appropriateness of the PYD-SF for Peruvian university students.

## Conclusion

This study provides evidence based on internal structure, internal consistency, measurement invariance across sex, the empirical distinctiveness of the five dimensions, and relations with purpose in life for the interpretation of PYD-SF scores in Peruvian university students. All five dimensions showed positive and statistically significant associations with purpose in life. These theoretically consistent associations constitute validity evidence based on relations with another variable, rather than a direct test of convergent validity.

Taken together, the findings support the use of PYD-SF scores for group-level research in comparable university populations. However, the present study does not establish content validity, temporal stability, predictive validity, responsiveness, norms, cut-off scores, or suitability for individual or clinical assessment. These additional sources of evidence should be examined before extending the interpretation or use of the scores beyond the purposes evaluated in this study.

Future studies should further examine alternative modelling approaches, including bifactor ESEM or exploratory structural equation modelling (ESEM), that more accurately represent the latent structure of the construct and address residual multidimensionality. It is also recommended that the developmental and cultural relevance of items with lower factor loadings be examined before considering any item revision. Finally, longitudinal and cross-cultural studies will allow for the examination of the temporal stability of the PYD and its characteristics in different sociocultural contexts in Latin America.

## Data Availability

The raw data supporting the conclusions of this article will be made available by the authors, without undue reservation.
