# Supplementary material for: Psychometric validation of the positive youth development scale–short form in Peruvian university students
Source: Front Psychol. 2026 Sep 30;17:1909136. doi: 10.3389/fpsyg.2026.1909136 (PMC13661508; doi:10.3389/fpsyg.2026.1909136)
Supplement: Supplementary file 1 [file Table_1.DOCX]

**Supplementary Figure S1.** Bifactor model of the Positive Youth Development Scale–Short Form (PYD-SF)

*Note*: Factor01 = Competence; Factor02 = Confidence; Factor03 = Character; Factor04 = Caring; Factor05 = Connection. The bifactor model includes one general Positive Youth Development (PYD) factor together with five specific factors representing Competence, Confidence, Character, Caring, and Connection. The same six residual covariances specified in the correlated five-factor model were retained. Standardized factor loadings are displayed on the arrows, and values correspond to standardized parameter estimates obtained using the DWLS estimator.

**Supplementary Table S1.** Individual explained common variance (IECV) for the bifactor model of the PYD-SF

| **Item code** | **IECV** |
| --- | --- |
| Item_D_01 | 0.509 |
| Item_D_02 | 0.954 |
| Item_D_03 | 0.632 |
| Item_D_04 | 0.608 |
| Item_D_05 | 0.512 |
| Item_D_06 | 0.432 |
| Item_D_07 | 0.996 |
| Item_D_08 | 0.925 |
| Item_D_09 | 0.487 |
| Item_D_10 | 0.777 |
| Item_D_11 | 0.849 |
| Item_D_12 | 0.633 |
| Item_D_13 | 0.629 |
| Item_D_14 | 0.975 |
| Item_D_15 | 0.772 |
| Item_D_16 | 0.514 |
| Item_D_17 | 0.561 |
| Item_D_18 | 0.621 |
| Item_D_19 | 0.501 |
| Item_D_20 | 0.533 |
| Item_D_21 | 0.583 |
| Item_D_22 | 0.294 |
| Item_D_23 | 0.057 |
| Item_D_24 | 0.118 |
| Item_D_25 | 0.135 |
| Item_D_26 | 0.105 |
| Item_D_27 | 0.798 |
| Item_D_28 | 0.440 |
| Item_D_29 | 0.729 |
| Item_D_30 | 0.870 |
| Item_D_31 | 0.474 |
| Item_D_32 | 0.485 |
| Item_D_33 | 0.903 |
| Item_D_34 | 0.999 |

Note. IECV = individual explained common variance. IECV represents the proportion of an item’s common variance attributable to the general factor in the bifactor model.
